# Intracranial Stenting After Failed Thrombectomy in Patients With Moderately Severe Stroke: A Multicenter Cohort Study

**DOI:** 10.3389/fneur.2020.00097

**Published:** 2020-02-14

**Authors:** Lukas Meyer, Jens Fiehler, Götz Thomalla, Lars Udo Krause, Stephan Lowens, Jan Rothaupt, Byung Moon Kim, Ji Hoe Heo, Leonard Yeo, Tommy Andersson, Christoph Kabbasch, Franziska Dorn, René Chapot, Christian Paul Stracke, Uta Hanning

**Affiliations:** ^1^Department of Diagnostic and Interventional Neuroradiology, University Medical Center Hamburg-Eppendorf, Hamburg, Germany; ^2^Department of Neurology, University Medical Center Hamburg-Eppendorf, Hamburg, Germany; ^3^Department of Neurology, Klinikum Osnabrück, Osnabrück, Germany; ^4^Department of Radiology, Klinikum Osnabrück, Osnabrück, Germany; ^5^Department of Intracranial Endovascular Therapy, Alfried-Krupp Hospital, Essen, Germany; ^6^Department of Radiology, Interventional Neuroradiology, Severance Stroke Center, Severance Hospital, Yonsei University College of Medicine, Seoul, South Korea; ^7^Department of Neurology, Severance Stroke Center, Severance Hospital, Yonsei University College of Medicine, Seoul, South Korea; ^8^Department of Neuroradiology, Karolinska Institutet, Karolinska University Hospital and Clinical Neuroscience, Stockholm, Sweden; ^9^Division of Neurology, Department of Medicine, National University Health System, Singapore, Singapore; ^10^Department Medical Imaging, AZ Groeninge, Kortrijk, Belgium; ^11^Department of Neuroradiology, University of Cologne, Cologne, Germany; ^12^Department of Neuroradiology, University Hospital of Munich, Munich, Germany

**Keywords:** failed thrombectomy, stroke, intracranial stenosis, stent, ICAD

## Abstract

**Background and Purpose:** Recently, acute intracranial stenting (ICS) has gained more interest as a potential bailout strategy for large vessel occlusions (LVO) that are refractory to thrombectomy. However, there are currently no reports on ICS in patients with moderately severe stroke discussing the question if implementing a permanent stent is feasible and leads to improved recanalization after failed thrombectomy.

**Methods:** We analyzed a large multicenter database of patients receiving ICS for anterior circulation LVO after failed thrombectomy. Inclusion criteria were defined as: Moderately severe stroke (National Institute Health Stroke Scale (NIHSS) ≤9 on admission), anterior circulation LVO, acute ICS after failed stent retriever MT. Primary endpoint was the rate of improved successful recanalization after ICS defined as a modified Thrombolysis In cerebral Infarction (mTICI) score≥2b. Favorable neurological outcome was defined as an early neurological improvement (ENI) of 4 points or reaching 0 with respect to baseline NIHSS.

**Results:** Forty-one patients met the inclusion criteria. A median of 2 retrievals were performed (IQR 1–4) prior decision-making for ICS. ICS led in 90.2% (37/41) of cases to a final mTICI≥2b with significant improvement (*p* < 0.001) after the last retrieval attempt. The median NIHSS decreased (*p* = 0.178) from 7 (IQR 3.5–8) on admission to 2.5 (IQR 0–8.25) at discharge. ENI was observed in 47.4% (18/38). sICH occurred in 4.8% (2/41).

**Conclusion:** ICS after failed thrombectomy appears to effectively improve recanalization rates in patients with moderately severe strokes. Thus, ICS should be considered also for patients with baseline NIHSS ≤9 if thrombectomy fails.

## Introduction

Mechanical thrombectomy (MT) has become the standard of care for acute ischemic stroke due to large vessel occlusions (LVO). However, up to 29% of all mechanical thrombectomies (MT) fail due to several conditions, such as intracranial atherosclerotic disease (ICAD), calcified wall-adherent thrombi, dissections, or other rare pathologies (1–3). Accordingly, the best currently available evidence for the endovascular treatment of ICAD is based on the SAMMPRIS and the VISSIT study (4, 5) showing the superiority of best medical treatment over elective intracranial stenting. Recently, acute intracranial stenting (ICS) has been reported to be a highly promising bailout strategy for theses frustrating thrombectomy cases with predictably poor outcomes (6–11). Since these cases are still rare, past retrospective studies mostly analyzed heterogeneous cohorts, including a wide range of stroke severities (12).

It has been suggested that a low NIHSS (National Institute Health Stroke Scale) score is a more frequent observation in patients with acute occlusions of preexisting ICAD, presumably based on adaptation of collaterals to the chronic low flow conditions (13, 14). However, these patients arriving with comparably low NIHSS scores could still have a poor prognosis and high risk for stroke recurrence without sufficient therapy (15, 16). Therefore, it might not be reasonable to base decision-making for bailout strategies after failed thrombectomy on patients' initial NIHSS score only.

This study analyzes ICS for patients with moderately severe anterior circulation LVO strokes (NIHSS score ≤ 9 upon admission) to better estimate potential risks and benefits. We hypothesize that in this patient subgroup, ICS is a feasible bailout strategy to achieve improved vessel recanalization after failed thrombectomy.

## Methods

### Patient Selection

**Figure 1:**
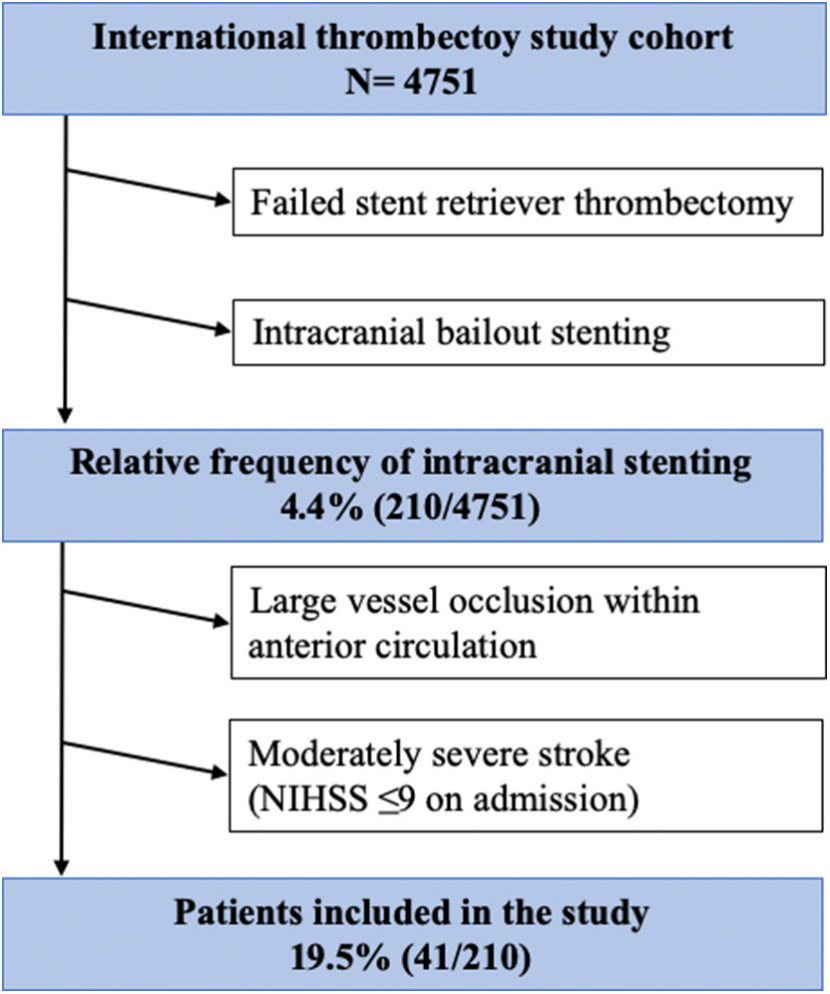
Flow chart of patient inclusion.

We analyzed all patients with moderately severe stroke from a large international ICS multicenter cohort (*n* = 4751) treated between 01/2014 and 12/2018. ICS after failed thrombectomy was performed in 210 cases with a relative frequency of 4.4 % (210/4751) in relation to all thrombectomies performed within the study period. Inclusion criteria were: (1) Moderately severe stroke (admission NIHSS ≤ 9), (2) anterior circulation LVO (3) MT performed exclusively with stent retrievers (4) acute intracranial stenting as a bailout strategy (Figure 1). The study was approved by the local ethics committee (Chamber of Physicians, Hamburg, Germany). Due to the retrospective and anonymized study design, informed consent of the patients was not required. Some data were part of previously published cohorts (7, 11).

### Baseline Characteristics

Available baseline characteristics were analyzed (Table 1). Early ischemic changes were graded with the Alberta Stroke Program Early CT Score (ASPECTS) on non-contrast computed tomography. Experienced neurologists examined all patients applying the NIHSS on admission and discharge. If eligible, all patients received intravenous thrombolysis (IVT) prior to MT.

**Table 1:** Overview of patients' baseline characteristics.

**Baseline characteristics**	**Study cohort (*n* = 41)**
Clinical and imaging	
Age (years), median (IQR)	64 (48–73)
Sex (men) % (n)	70.7 (29/41)
ASPECTS, median (IQR)	8 (8–10)
NIHSS on admission, median (IQR)	7 (3.5–8)
Pre-stroke mRS, % (n)	
0	65.9 (27/41)
1	26.8 (11/41)
2	4.9 (2/41)
3	2.4 (1/41)
Target vessel, *n* (%)	
ICA	36.6 (15/41)
M1	53.6 (22/41)
M2	9.8 (4/41)
Procedural	
Intravenous thrombolysis, % (n)	61 (25/41)
CT to groin-puncture (minutes), median (IQR)	104 (74.0–148)
Type of stent, % (n)	
Self-expandable	100 (41/41)

### Intervention

In all cases MT performed with approved stent retriever devices did not lead to sufficient recanalization or direct reocclusion after thrombectomy occurred. Accordingly, the number of retrieval attempts as well as the moment of decision-making for ICS and declaring thrombectomy as failed was left to the interventionalist. All types of stent retriever, the number of thrombectomy maneuvers, as well as the stent design (balloon or self-expanding) were evaluated. The recanalization result was evaluated with the modified thrombolysis in cerebral infarction (mTICI) score.

### Procedural and Functional Outcome

Primary endpoint was the rate of improved recanalization after ICS assessed by the rate of successful recanalization defined as mTICI≥2b. Neurological outcome was assessed by the rate of early neurological improvement (ENI) defined as a decrease in NIHSS at discharge from baseline of at least 4 points as previously described (17) or reaching 0. The rate of favorable functional outcome was assessed as mRS≤2 at 90 days. Due to the retrospective approach, ENI data for 3 patients and 90 day mRS data for 14 patients were missing. For safety assessment, cases with symptomatic intracranial hemorrhage (sICH) according to ECASS-II (18), mortality, and intervention related complications were evaluated. Further, the antiplatelet therapy regimes were recorded and analyzed.

### Statistical Analysis

Continuous variables are displayed as mean with SD or median with interquartile range. For categorical data, absolute, and relative frequencies are displayed. Wilcoxon test was performed to compare stroke severity on admission and discharge, as well as recanalization status before and after stenting. *P* ≤ 0.05 were considered significant. Analyses were performed using SPSS V.25 (IBM Corporation, Armonk, New York, USA).

## Results

### Baseline Characteristics

19.5% (41/210) of all patients in the multicenter ICS database met the required inclusions criteria. Median age was 64 years (IQR 48.5–73.5) and 70.7% (29/41) were men. On admission median ASPECTS was 8 (IQR 8–10) and median 7 (IQR 3.5–8), whereas the median pre-stroke mRS was 0 (IQR 0–1). 39% (16/41) of the patients received IVT prior to MT. Target vessels for ICS were ICA in 36.6% (15/41), MCA M1 in 53.7% (22/41) and MCA M2 in 9.8% (4/41). A self-expandable stent (Acclino flex®, Neuroform®, Solitaire®, Enterprise®, Wingspan®) was utilized in all cases (100%, 41/41) for bailout ICS. Information on periprocedural antithrombotic medication was available in 51.1% (21/41). In 14.3% (3/21) IV acetylsalicylic acid only and in 85.7% (18/21) glycoprotein-IIb/IIIa-antagonists were administered. Post-interventionally, all patients received dual antiplatelet therapy for 3 months. Table 1 gives an overview of patients' baseline characteristics.

### Procedural and Functional Outcome

**Figure 2:**
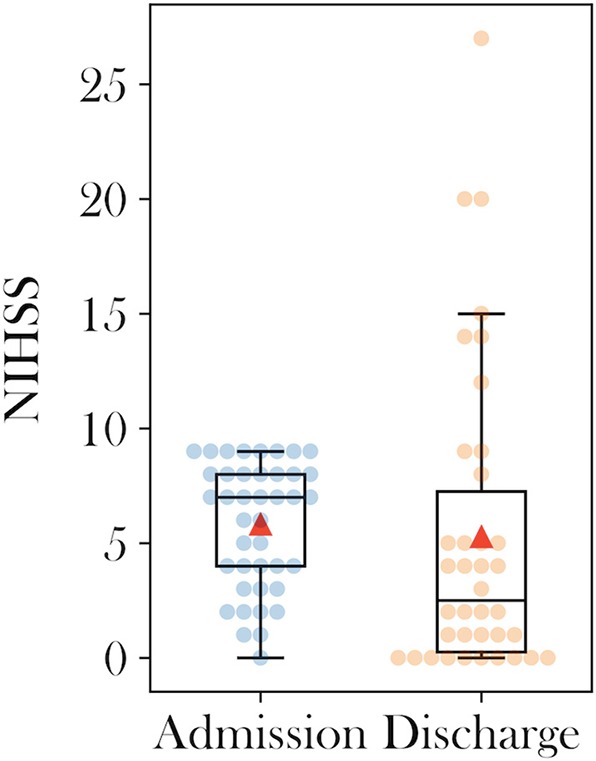
Admission and discharge boxplots of National Institutes of health stroke scale scores.

A median of 2 MT maneuvers were performed (IQR 1–4) prior to ICS. After the final MT attempt mTICI≥2b was achieved in 43.9% (18/41). Acute ICS significantly increased (*p* < 0.001) the rate of mTICI≥2b to 90.2% (37/41). The median NIHSS decreased from 7 (IQR 3.5–8) on admission to 2.5 (IQR 0–8.25) at discharge (Figure 2) without reaching statistical significance (*p* = 0.178). ENI was observed in 47.4% (18/38) and sICH occurred in 4.8% (2/41) of all patients. At 90 days the rate of mRS≤2 was 74.1% (20/27; Table 2) and the mortality was 2.7% (1/27).

**Table 2:** Overview of procedural results, neurological and functional outcome, complications, and mortality.

**Results**	**All patients (*n* = 41)**
Procedural	
Passes of retriever, median (IQR)	2 (1–4)
Successful recanalization after last pass	43.9 (18/41)
(mTICI 2b/3), *n* (%)	
Successful recanalization after ICS	90.2 (37/41)
(mTICI 2b/3), *n* (%)	
Neurological improvement	
NIHSS discharge, median (IQR)	2.5 (0–8.25)
ENI at discharge % (n)	47.4 (18/38)
Complication	
sICH % (n)	4.8 (2/41)
Functional outcome	
mRS ≤2 at 90 days % (n)	74.1 (20/27)
Mortality	
At 90 days % (n)	2.7 (1/27)

## Discussion

Recent retrospective case series have suggested reconsidering ICS as a bailout strategy when MT fails to recanalize LVOs (11, 19). All these ICS studies report on typical thrombectomy cohorts with a median admission NIHSS ranging from 14 to 19 (12). However, there are currently no reports on ICS after failed thrombectomy for a homogenous group of less severe stroke patients. Accordingly, we defined strokes with a baseline NIHSS ≤ 9 on admission as moderately severe. This study assesses a potential benefit of ICS after failed thrombectomy for this subgroup.

Successful recanalization is a strong predictor for long-term favorable functional outcome (mRS≤2) after MT (20). In cases with ICAD, acute or prolonged reocclusion after MT is a severe complication leading to poor outcomes (21). Even with supposedly higher proportions of low NIHSS on admission in patients with ICAD due to possibly better adapted collateral flow, a sufficient and sustainable vessel recanalization seems crucial to increase chances for long-term favorable outcomes and to prevent stroke recurrence (14). Thus, it is an important finding in our study that ICS increased the rate of successful recanalization leading to 90% mTICI≥2b after failed MT attempts.

ENI was observed in 47% of the cohort with a median NIHSS decrease of 4.5 points from admission to discharge. This finding suggests a neurological benefit of ICS in our cohort, however the prespecified level of statistical significance was not reached most likely due to the underpowered sample size of the study. Furthermore, the 90 days favorable functional outcome (mRS≤2) was above average (74.1%, 20/27) of past thrombectomy landmark studies (1). This finding highlights both, the limitations of the retrospective study design with missing follow-up data and the strength of the finding, showing that even if poor outcome had been observed in all 14 missing patients, the final rate of favorable outcome would still have been comparably good with 49% of mRS≤2 (20/41).

The necessity of antiplatelet therapy after permanent stenting has always been a major concern in endovascular stroke treatment due to its increased risk for intracerebral bleeding (22). Choosing ICS for patients presenting with low NIHSS might be of even higher concern since the risk-benefit balance is not comparable to a patient with a high baseline NIHSS facing a high risk of poor outcome. In our study on ICS for moderately severe stroke, 2/41 patients experienced sICH. This result is comparable to those in the HERMES meta-analysis with 4.4% and is unexpectedly low compared to the latest acute ICS studies that reported on sICH rates ranging from 8 to 17% (1, 12). Antithrombotic medication was administered periinterventionally in all our cases, some even in combination with IVT. Even though detailed information were only available in 50% of all patients, our finding is in line with the latest studies observing the safety of stenting combined with antiplatelet therapy in the setting of acute tandem occlusions (23, 24).

Even though we do not have data on stent patency in this cases series due to missing follow-up imaging, Chang et al. (19) previously observed that a favorable 90 day outcome (mRS≤2) is significantly associated with stent patency. The median number of retrieval attempts in our study was 2. Since all cases were performed in tertiary stroke centers with expertise in all kinds of neurointerventions, this finding is consistent with the latest reports on risk-benefit ratio of additional retrieval attempts and encourages to perform acute ICS after a maximum of three thrombectomy maneuvers even in patients with moderately severe strokes (20, 25).

## Limitations

Our study has all limitations that come along with a retrospective study design. Major limitations are the aforementioned missing data on antithrombotic medication and follow-up outcome at 90 days in 14 patients as well as a control group treated with IVT only for comparison of clinical efficacy and safety endpoints.

## Conclusion

This study with its focus on moderately severe stroke is in line with recently published articles that suggested the feasibility of ICS as a bailout strategy after failed thrombectomy leading to improved recanalization in the endovascular treatment of acute ischemic stroke.

## Data Availability Statement

The data that support the findings of this study are available from the corresponding author upon reasonable request.

## Ethics Statement

The studies involving human participants were reviewed and approved by Chamber of Physicians, Hamburg, Germany. Written informed consent for participation was not required for this study in accordance with the national legislation and the institutional requirements.

## Author Contributions

LM, UH, CS, and JF: conception and design of the study. LM, UH, GT, CK, LY, FD, TA, BK, SL, JR, JH, and LK: acquisition and analysis. LM, UH, RC, FD, TA, BK, LY, CS, and JF: drafting and revising the manuscript critically.

### Conflict of Interest

JF received research support from German Ministry of Science and Education (BMBF), German Ministry of Economy and Innovation (BMWi), German Research Foundation (DFG), European Union (EU), Hamburgische Investitions- und Förderbank (IFB), Medtronic, Microvention, Philips, Stryker, Consultant for: Acandis, Boehringer Ingelheim, Cerenovus, Covidien, Evasc Neurovascular, MD Clinicals, Medtronic, Medina, Microvention, Penumbra, Route92, Stryker, Transverse Medical. GT received consulting fees from Acandis, grant support and lecture fees from Bayer, lecture fees from Boehringer Ingelheim, Bristol-Myers Squibb/Pfizer, and Daiichi Sankyo, and consulting fees and lecture fees from Stryker. LK received speaker honoraria from Boehringer Ingelheim, Medtronic and JR is consultant for Acandis and Phenox. TA is a consultant for Ablynx, Amnis Therapeutics, Medtronic, Cerenovus/J&J, Rapid Medical and Anaconda. LY has received substantial grant funding from the National Medical Research Council (NMRC), Singapore and substantial support from the ministry of health (MOH), Singapore. CK is proctor for Acandis. FD is consultant for Acandis. RC is consultant and/or proctor for BALT, Stryker, Microvention, Rapid Medical, Siemens Medical Systems. CS is consultant and/or proctor for Acandis, Balt, and Rapid Medical. The remaining authors declare that the research was conducted in the absence of any commercial or financial relationships that could be construed as a potential conflict of interest.

## Abbreviations

ASPECTS: Alberta Stroke Program Early CT Score
ENI: Early Neurological Improvement
LVO: Large Vessel Occlusion
MT: Mechanical Thrombectomy
mTICI: Modified Thrombolysis In cerebral Infarction
NIHSS: National Institute Health Stroke Scale
ICS: Intracranial Stenting
ICAD: Intracranial Atherosclerotic Disease
IVT: Intravenous Thrombolysis
sICH: symptomatic Intracerebral Hemorrhage.
